# Troxipide in the Management of Gastritis: A Randomized Comparative Trial in General Practice

**DOI:** 10.1155/2010/758397

**Published:** 2010-11-25

**Authors:** Bhupesh Dewan, Aarthi Balasubramanian

**Affiliations:** Medical Services Department, Zuventus Healthcare Ltd, 5119, ‘D' Wing, Oberoi Garden Estates, Chandivali, Mumbai 400072, India

## Abstract

*Background*. A trial of empirical acid-suppressive therapy is the usual practice for most patients with symptoms of gastritis in primary care. *Aim*. To assess the relative efficacy of Troxipide and Ranitidine in patients with endoscopic gastritis over a four-week period. *Methods*. In all, 142 patients were randomized to Troxipide (100 mg tid) or Ranitidine (150 mg bid) for a period of four weeks. The severity of the signs of endoscopic gastritis at baseline and week 4 using a four-point scale and the subjective symptom severity at baseline and week 2 & week 4 using a Visual analog scale (VAS) were documented. *Results*. Troxipide was found to be superior to Ranitidine for both, the complete resolution and improvement of endoscopic gastritis. Higher proportion of patients showed complete healing of erosions (88.14%), oozing (96.77%), and edema (93.88%) with Troxipide as compared to Ranitidine (*P* < .01). Patients receiving Troxipide also showed a greater improvement in the VAS scores for abdominal pain, bloating, and heartburn (*P* < .01). Both the drugs were found to be well tolerated. *Conclusion*. In patients with endoscopic gastritis, Troxipide, with its superior rate of improvement, resolution of signs, and subjective clinical symptoms, can be considered as an alternative to the commonly used antisecretory agents.

## 1. Introduction

Gastrointestinal disorders like gastritis are highly prevalent diseases among the Asian population [1], with majority of the patients approaching their general practitioners only after the disease-related symptoms become more frequent or more severe. These symptoms have been known to cause marked disruption of physical, social, and emotional well-being of the patients [2], and, hence, therapy is generally directed at controlling these symptoms quickly and efficiently.

Gastric antisecretory agents like the histamine-2-receptor antagonists (H2RAs), proton-pump inhibitors (PPIs), and the Cytoprotective agents (antacids, sucralfate) have been successfully used for years in the treatment of gastrointestinal disorders like gastritis [3]. Despite many efforts, the pharmacological treatment of patients with gastritis using acid suppressants usually achieves only partial symptomatic relief in the majority of the cases [4]. 

Of late, however, the role of the cytoprotective agents in strengthening the mucosal defensive factors is gaining importance. It is now assumed that these drugs ultimately balance the aggressive factors (acid, pepsin, H. pylori, and bile salts) and defensive factors (mucin secretion, cellular mucus, bicarbonate secretion, mucosal blood flow, and cell turnover) [5–7]. Although these drugs have brought about remarkable changes in ulcer therapy, the efficacy of these drugs is still debatable.

Troxipide is a new gastric cytoprotective agent, which neither inhibits acid secretion nor has acid neutralizing activity, but has been clinically proven to heal gastritis and gastric ulcers [8–10]. Troxipide has shown to inhibit neutrophil mediated inflammation [11] and oxidative stress [11–15] in addition to improving the gastric mucus composition and output [9, 16]. Furthermore, it has been found to increase the secretion of cytoprotective prostaglandins [10]. The gastric mucosal metabolism [17] and blood flow [18] are also enhanced by Troxipide. Nearly 60% of gastric ulcers were reported to be completely healed within 8 weeks of the administration of 300 mg/day of Troxipide [11].

Although preclinical data have found Troxipide to be a more efficacious agent than the prototype cytoprotectants [13, 17, 19] and acid suppressants (famotidine and ranitidine) [20], there is limited clinical data on the comparative efficacy of Troxipide with other therapeutic agents used in the treatment of gastrointestinal disorders. This clinical study has been conducted to compare the relative efficacy of a cytoprotective agent, Troxipide, with an acid suppressive agent, Ranitidine, in alleviating, over a four-week period, the symptoms of gastritis.

## 2. Methods

This multicentric, randomized therapeutic trial was planned to compare the effects of Troxipide and Ranitidine on the resolution of endoscopically proven gastritis for a period of four weeks. The study was conducted in accordance with the Declaration of Helsinki and its subsequent revisions and as per the ICH Good Clinical Practice (GCP) guidelines. The Central Independent Ethics Committee-Clinical Research, New Delhi, India approved the protocol of the study.

### 2.1. Patients

Patients (between 18–65 yrs), referred for upper gastrointestinal endoscopy, were recruited from the outpatient clinics of five hospitals across India. The primary requirement for inclusion was the presence of endoscopic gastritis, as diagnosed by the Sydney classification. The main exclusion criteria included presence of perforation or pyloric stenosis, esophageal stricture or intestinal obstruction, previous history of gastrointestinal disease (inflammatory bowel disease, malabsorption syndromes, gastrointestinal malignancy), recent gastrointestinal surgery, that is, within 30 days (vagotomy, Barrett's esophagus and scleroderma), prior administration of PPIs, H_2_RAs, prokinetic agents or any other gastroprotective agent within 7 days of screening, and a known history of hypersensitivity to study medications.

### 2.2. Study Design

After obtaining the informed consent, all the patients underwent a complete physical examination, and relevant demographic details were noted. Laboratory investigations, which included complete blood count, hemoglobin, occult blood and serology (ELISA) for Helicobacter pylori, and upper gastrointestinal endoscopy, were carried out. The patients were then randomized, based on a computer-generated randomization sequence, to either the Troxipide treatment arm or the Ranitidine treatment arm. They received either Troxipide 100 mg orally thrice daily or Ranitidine 150 mg orally twice daily for a period of 28 days.

General systemic and laboratory investigations were performed before the administration of the study medication. Followup visits were scheduled after week 2 and week 4 of therapy. 

The patient's medication supply was divided into two parts, administered at the time of enrollment and on day 14 (after week 2). Thus each investigator was provided with medication supply for 50 patients: plastic containers containing either 45 tablets of Troxipide or 30 tablets of Ranitidine for each patient.

### 2.3. Assessments

The topography and severity of endoscopic gastritis was classified according to the Sydney System of Endoscopic Classification [21, 22]. Based on the endoscopy, the topography was noted as antrum, corpus, or both (pangastritis). The severity of the findings from the upper gastrointestinal endoscopy (carried out at baseline and week 4) were rated on a four-point scale: 1 (no erosion/absent), 2 (1–3 erosions/mild), 3 (4–6 erosions/moderate), and 4 (more than 6 erosions/severe) [4]. 

The visual analog scale (VAS), a scoring system from 0 (lack of symptom) to 100 (high severity) [23], was used to score the severity of the seven subjective clinical symptoms of gastritis (abdominal pain, bloating, belching, nausea, vomiting, loss of appetite, and heartburn) at baseline and each follow up visit. These severity score were further graded as: none (VAS score 0), mild (VAS score 1–30), moderate (VAS score 31–60), and severe (VAS score 61–100) [24].

The patients and investigators were asked to rate the tolerability of the study medications on a four-point scale: 0 (very good), 1 (good), 2 (fair), and 3 (poor).

Adverse events, if any, were reported during the followup visits.

### 2.4. Outcome Measures

There were two primary outcome measures considered, namely, the proportion of patients achieving complete resolution of the endoscopic gastritis (reduction of four-point scale score for individual endoscopic signs to 0 at end of therapy), and symptom relief (proportion of patients achieving a reduction in VAS score of at least 50 for the various clinical symptoms of gastritis studied from baseline to week 2 and week 4). The other secondary outcome measures were proportion of patients showing improvement in endoscopic gastritis (a reduction of severity of the baseline four-point scale score for individual endoscopic findings at the end of week 4), the proportion of patients showing VAS score of 0 at end of week 2 and week 4 (Complete Symptomatic Resolution), the proportion of patients showing a reduction of greater than 20 points in VAS scores from baseline to week 2 and week 4 (Symptom Improvement), and Treatment Tolerability.

### 2.5. Statistical Analysis

The sample size was calculated based on the assumption that a difference of 20% in the control group would be detected with a responder rate of 80% in the test group and a dropout rate of 25%. Thus, a sample of 100 completed patients in each group was found to be sufficient at 5% level of significance and 80% power.

The paired Students *t*-test or Wilcoxon ranks test was used to analyze the intraindividual differences between baseline and posttreatment values while the intertreatment differences were analyzed with the unpaired Student's *t*-test (when means were considered) or Fisher's exact test (when proportions were considered). All data are presented as mean ± standard deviation (SD) unless stated otherwise. *P* value less than.05 was considered significant.

## 3. Results

One hundred and forty-four patients with symptomatic gastritis or dyspepsia were randomized into two groups; 72 patients received Troxipide while 72 patients received Ranitidine. In two patients, one from each group, no followup data were available; they withdrew from the trial after baseline evaluations.

### 3.1. Patient's Profile

The demographic and baseline clinical characteristics of the 142 patients (71 in the Troxipide group and 71 in the Ranitidine group) included in the study are given in Table 1. Baseline systemic examinations were normal in all patients; however, gastrointestinal system examination was found to be abnormal in 8.45% (12/142) of the patients. 

A history of gastritis and/or gastroesophageal reflux disorders (GERD) was reported in 13.38% (19/142) of the patients (10 in the Troxipide group and 9 in the Ranitidine group) while prolonged therapy with nonsteroidal anti-inflammatory drugs (NSAIDs) was reported in one patient randomized to the Troxipide group. A total of 9.86% (14/142) of the patients used concomitant medications like domperidone, lactulose, and nystatin.

### 3.2. Gastroendoscopic Findings

As per the diagnosis by endoscopy, the signs of gastritis were limited to the antrum in 34.51% of the patients and limited to the corpus in 9.15% of the patients while pangastritis was found in 54.93% of the patients (Table 1). The endoscopic examination also revealed that gastritis was either idiopathic (84.5%) or was associated with the probable presence of H. pylori (7.75%), the usage of drugs (4.23%), or other gastric irritants (3.52%). However, among the patients showing the probable endoscopic presence of H. pylori, only two patients (both from Troxipide group) had a corresponding positive serology.

### 3.3. Complete Resolution of Signs of Endoscopic Gastritis

Among the 142 patients, a higher proportion of patients receiving Troxipide showed complete endoscopic healing of erosions (88.14% versus 56.36%), oozing (96.77% versus 78.95%), and edema (93.88% versus 46.51%) as compared to Ranitidine (*P* < .05). 

Complete endoscopic healing was also found to be higher with Troxipide than Ranitidine among the patients showing moderate to severe signs of endoscopic gastritis—85.71% versus 41.66% for erosion, 71.43% versus 34.29% for redness and 92% versus 41.18% for edema (*P* < .01).

Twenty-seven of the patients receiving Troxipide and seventeen of those receiving Ranitidine showed the presence of all four endoscopic signs at baseline. Of these patients, complete endoscopic healing was seen in 77.77% of the patients (21/27) receiving Troxipide and 29.41% (5/17) of those receiving Ranitidine (*P* < .01; 95% CI: 16.8–79.9).

### 3.4. Improvement in Signs of Endoscopic Gastritis

The reduction in the mean severity scores of the various endoscopic findings from baseline and week 4 were greater with Troxipide than Ranitidine as seen in Table 2.

An improvement in the endoscopic gastritis was also found in a greater proportion of patients receiving Troxipide: gastric mucosal erosion (98.31% versus 78.18%), oozing (97.77% versus 78.95%), redness (91.04% versus 71.43%), and edema (97.96% versus 69.77%) as compared to Ranitidine at the end of therapy (*P* < .05).

### 3.5. Clinical Symptom Relief

At the end of therapy duration, a higher proportion of patients reported symptom relief (reduction of at least 50 points on the VAS score from baseline to follow up) with Troxipide: abdominal pain (70.42% versus 19.72%), bloating (44.78% versus 18.46%), belching (43.18% versus 20.51%), and heartburn (56.90% versus 16.33%) as compared to Ranitidine (*P* < .05). It was also found that all these patients had moderate to severe endoscopic gastritis at baseline.

### 3.6. Complete Symptom Resolution

**Figure 1:**
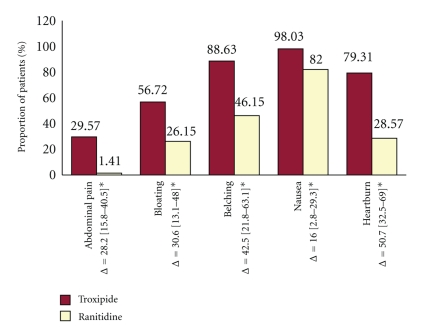
Complete symptom resolution in endoscopic gastritis with Troxipide and Ranitidine. Percentage of patients at the end of therapy (week 4) reporting complete symptom resolution (a VAS score of 0 at week 4) with Troxipide and Ranitidine at the end of treatment (*P < .01).

Complete resolution of the subjective symptoms reported at baseline was reported in a statistically greater proportion of patients receiving Troxipide as compared to Ranitidine (*P* < .01) at the end of week 4 as seen in Figure 1.

### 3.7. Improvement in Clinical Symptoms

**Figure 2:**
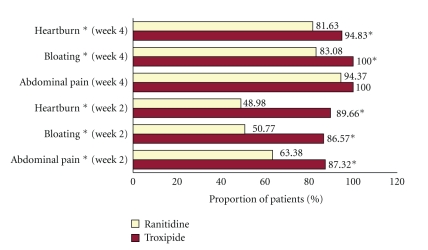
Clinical symptom improvement in endoscopic gastritis with Troxipide and Ranitidine. Percentage of patients at each followup period (week 2 and 4) reporting clinical symptom improvement (a reduction in the VAS score of at least 20 points from baseline to followup) for abdominal pain, bloating, and heartburn with Troxipide and Ranitidine (*P < .05 and **P < .01).

Some positive change in the severity of the subjective symptom was seen in all the 142 patients participating in the study. However, the reduction in the mean VAS score, at the end of week 2 and week 4, were found to be superior with Troxipide as compared to Ranitidine (Table 3), especially for abdominal pain, bloating, and heartburn (*P* < .01). Symptoms improvement (a reduction in the VAS score of at least 20 points from baseline to followup) was seen in a higher proportion of patients with Troxipide as compared to Ranitidine in case of abdominal pain, bloating, and heartburn (Figure 2).

### 3.8. Overall Safety and Tolerability Profile

Mild to moderate cases of constipation and headache were reported among fourteen patients (four receiving Troxipide and ten receiving Ranitidine). However, these adverse effects did not warrant discontinuation of the treatment and the overall tolerability profile of the study medications was not affected.

The investigators and the patients also found Troxipide to be a better tolerated drug than Ranitidine. A higher mean proportion of patients and investigators rated its tolerability as very good (77.46% versus 21.13%) or good (21.84% versus 31.69%).

## 4. Discussion

Gastrointestinal diseases such as gastritis have a significant impact on healthcare. As a result, healthcare providers must devise ways to limit the expenditure while providing high-quality patient care. In case of gastritis treatment, the efficacy of the medication to be used should be considered, because recurrence and complications resulting from ineffective therapies can negatively affect the patient's quality of life and increase the overall cost of healthcare [25]. Studies have shown that patients receiving at least one prescription of acid-suppressive agents had a substantial increase in the risk of developing pancreatitis [26]. It has therefore been suggested that cytoprotective drugs, which ultimately balance the aggressive and defensive factors, may be the more appropriate means of controlling the disease [5, 7].

The present study, comparing Troxipide (100 mg thrice a day) with Ranitidine (150 mg twice a day), has demonstrated the role of Troxipide as an effective prophylactic in patients with endoscopic gastritis. Troxipide was found to bring about an improvement in the severity of the signs of endoscopic gastritis and various subjective clinical symptoms in nearly 96.27% and 81.23% of the patients, respectively. The VAS scale used in this study is a validated outcome measure and has been used successfully in many earlier studies [23, 24, 27]. In the validation of the VAS scale, a 20-point change has been recommended as the benchmark of a clinically significant response [28, 29]. 

In patients with moderate to severe endoscopic gastritis, Troxipide was more effective than ranitidine in bringing about complete endoscopic healing and clinical symptom relief. The overall tolerability profile as assessed by the patients and investigators was also higher with Troxipide.

One of the cardinal signs of GERD [30, 31], heartburn, was considered as one of the important subjective symptoms reported by patients. It is interesting to note that Troxipide not only provides a higher proportion of patients with an improvement in heartburn, but also provides it in a shorter duration as compared to Ranitidine. This was evident from the fact that by the second week, the patients receiving Troxipide reported statistically comparable results to those produced by Ranitidine after four weeks of therapy. It can be concluded, therefore, that Troxipide may prove to be an efficient agent in the treatment of GERD.

The other major finding in this study is that the rate of prevalence of H. pylori infection in this series was exceptionally low, considering the study sample consisted of a series of patients referred for endoscopy. It is also striking that while the presence of H. pylori in the Indian population is substantial [32, 33], only 3.5% (5/142) of the study sample showed positive serology for H. pylori. The literature search for a few Indian studies also revealed very low incidence of 5.8%–17.7% of H. pylori infection among the patients showing the endoscopic presence of gastritis [34, 35]. In the earlier studies, the presence of H. pylori in the study population has generally been confirmed by subjecting the biopsy sample to the rapid urease test, in addition to either serology or the urease breath test [36, 37]. Additionally, the use of different cutoff points for the test positivity in different studies may lead to various sensitivities and specificities. Thus, the incidence of H. pylori infection can vary considerably among studies because of the selection of the diagnostic test as well as the cutoff points for test positivity [38]. 

It is also known that additional investigations and invasive procedures are required in patients only if the presence of H. pylori has been identified along with the presence of gastritis or dyspepsia related symptoms [39]. In the present study, however, the histological aspects of endoscopic gastritis were not considered as part of the protocol. The inclusion of a provision to extract biopsy samples for the rapid urease test could probably have revealed the presence of H. pylori in more patients.

Current therapy focuses on eradication of H. pylori in addition to the healing of gastritis. The Maastricht III Consensus Report [40] also recommends a treatment for weeks with triple eradication therapy, irrespective of the geographical region, which would ultimately results in the complete resolution of gastritis. However, studies in the Indian population have found that rate of eradication of H. pylori using this triple therapy for one week has been lower than those reported in the West [35, 41]. Troxipide may aid in the improvement of this eradication rate, either as a monotherapy or in combination with other currently used agents.

Although not significant, a small subgroup of the current study sample has shown complete eradication of H. pylori and subsequent healing of endoscopic gastritis after four weeks of therapy with Troxipide. This is further supported by earlier preclinical studies where Troxipide has been shown to inhibit H. pylori derived urease, fMLP-like substances, and myeloperoxidase activity as well as interleukin-8-induced chemotaxis [11]. There is a need, however, for further studies to evaluate the effectiveness of Troxipide in patients with H. pylori infection.

Studies evaluating the efficacies of cytoprotective agents in the treatment of nonulcer dyspepsia and nonerosive gastritis have generally found them to be equally or marginally more efficacious than placebo [42, 43]. Interestingly, randomized clinical trials comparing cytoprotective agents with the acid-suppressive agent, Ranitidine, have observed superior efficacy with the former [44, 45]. These studies have found that the global relief in symptoms is significantly more frequent with the cytoprotective agent than Ranitidine [44]. Further, the cytoprotective agent was more effective than ranitidine in inducing healing or improvement of the endoscopic and histological features of the disease [45]. The present study has also reported, in accordance to these earlier studies, a better therapy outcome with Troxipide as compared to Ranitidine.

Troxipide, in particular, has been extensively studied in patients with gastritis and gastric ulcer [8–10]. Pooled data from studies evaluating the efficacy of Troxipide in over 300 patients with gastritis or acute gastric mucosal lesions of chronic gastritis have demonstrated an overall amelioration rate of 82.9% [9]. Similarly, an amelioration rate of 79.4% has been reported among 514 patients with gastric ulcer [9]. The findings of the current study, using an indigenously prepared Troxipide sample [46], are in accordance with these earlier studies.

In addition to superior efficacy, the tolerability of the medication will have an impact on its compliance among the patient population. In the present study, patients found Troxipide to be better tolerable than Ranitidine, which in addition to its good efficacy profile makes Troxipide a suitable prophylactic agent.

This study demonstrates that Troxipide (300 mg) is more effective than Ranitidine (300 mg) in controlling the subjective symptoms and endoscopic findings in patients with moderate to severe gastritis in the primary care setting. Thus, it establishes the appropriateness of a Troxipide-based treatment strategy for primary care practice patients.

## Figures and Tables

**Table 1:** Baseline demographic and clinical characteristics of study population.

	Troxipide (*n* = 71)	Ranitidine (*n* = 71)
Sex (*n*, %)		
Male	46 (64.78)	35 (49.30)
Female	25 (35.21)	36 (50.70)
Age (yrs), mean (±SD)	36.82 ± 12.42	33.19 ± 10.79
Body mass index (kg/m^2^), mean (±SD)	24.12 ± 4.49	23.95 ± 4.33
Positive H. pylori urea breath test	5 (7.04)	0 (0.0)
Endoscopic site of Gastritis (*n*, %)		
Antrum	19 (26.76)	30 (42.25)
Corpus	7 (9.86)	6 (8.45)
Antrum and Corpus (Pangastritis)	45 (63.38)	33 (46.47)
Endoscopic Evidences (*n*, %)		
Erosion	59 (83.1)	55 (77.46)
Oozing	31 (43.66)	19 (26.76)
Redness	67 (94.36)	63 (88.73)
Edema	49 (69.01)	43 (60.56)
Gastritis clinical symptoms (*n*, %)		
Abdominal Pain	71 (100.0)	71 (100.0)
Bloating	67 (94.36)	65 (91.55)
Belching	44 (61.97)	39 (54.93)
Nausea	51 (71.83)	50 (70.42)
Vomiting	14 (19.72)	13 (18.31)
Loss of appetite	29 (40.85)	22 (30.99)
Heartburn	58 (81.69)	49 (69.01)

**Table 2:** Mean four-point scale (FPS) scores for signs of endoscopic Gastritis in patients at baseline and week 4.

	Troxipide mean VAS score (±SD)	Ranitidine mean VAS score (±SD)	*P* values for difference between treatments
Gastric Mucosal Erosion			
Baseline	2.58 ± 0.95	2.17 ± 0.84	
Week 4	1.08 ± 0.33*	1.39 ± 0.57*	<.01
Gastric mucosal Oozing			
Baseline	1.55 ± 0.71	1.31 ± 0.55	
Week 4	1.01 ± 0.21*	1.06 ± 0.23*	<.05
Gastric mucosal Redness			
Baseline	2.73 ± 0.72	2.45 ± 0.79	
Week 4	1.28 ± 0.48*	1.65 ± 0.63*	<.05
Gastric mucosal Edema			
Baseline	2.10 ± 0.91	1.87 ± 0.84	
Week 4	1.04 ± 0.26*	1.32 ± 0.53*	<0.05

*P Value less than  .05 versus baseline, within the group.

**Table 3:** Mean Visual Analog Scale (VAS) scores for Gastritis symptoms in patients at baseline, week 2 and week 4.

	Troxipide Mean VAS Score (±SD)	Ranitidine Mean VAS Score (±SD)	*P*-values for difference between treatments
Abdominal Pain			
Baseline	61.55 ± 11.66	56.47 ± 10.97	
Week 2	34.78 ± 10.67*	37.04 ± 10.87*	<.01
Week 4	10.98 ± 9.43*	22.67 ± 9.70*	<.01
Bloating			
Baseline	52.98 ± 16.61	48.15 ± 18.94	
Week 2	27.01 ± 13.93*	31.38 ± 15.49*	<.01
Week 4	7.76 ± 10.56*	16.92 ± 14.78*	<.01
Belching			
Baseline	43.41 ± 20.11	42.58 ± 17.83	
Week 2	20.23 ± 16.21*	23.84 ± 14.43*	N.S
Week 4	2.04 ± 6.32*	10.51 ± 13.16*	<.05
Nausea			
Baseline	32.74 ± 18.01	32.42 ± 15.82	
Week 2	9.61 ± 11.48*	14.6 ± 13.58*	N.S
Week 4	0.19 ± 1.40*	3.4 ± 8.72*	N.S
Vomiting			
Baseline	17.85 ± 14.23	20.0 ± 11.54	
Week 2	5.0 ± 11.6*	3.84 ± 5.06*	N.S
Week 4	0.0*	0.76 ± 2.77*	N.S
Loss of Appetite			
Baseline	32.75 ± 19.25	30.91 ± 19.97	
Week 2	10.34 ± 10.85*	13.63 ± 10.02*	N.S
Week 4	0.34 ± 1.85*	0.91 ± 2.94*	N.S
Heartburn			
Baseline	49.31 ± 15.32	42.85 ± 16.58	
Week 2	21.37 ± 11.15*	26.73 ± 12.81*	<.01
Week 4	2.75 ± 6.15*	11.63 ± 9.5*	<.01

*P Value less than  .01 versus baseline, within the group.

NS: not significant (*P* > .05).
